# Predictive modelling: parents’ decision making to use online child health information to increase their understanding and/or diagnose or treat their child’s health

**DOI:** 10.1186/1472-6947-12-144

**Published:** 2012-12-10

**Authors:** Anne M Walsh, Melissa K Hyde, Kyra Hamilton, Katherine M White

**Affiliations:** 1 School of Nursing, Institute of Health and Biomedical Innovation (IHBI), Queensland University of Technology, GPO Box 2434, Brisbane, Queensland, 4001, Australia; 2 Behavioural Basis of Health, Griffith Health Institute and School of Applied Psychology, Griffith University, 176 Messines Ridges Road, Mt Gravatt, Queensland, 4122, Australia; 3 School of Applied Psychology, Griffith University, 176 Messines Ridges Road, Mt Gravatt, Queensland, 4122, Australia; 4 School of Psychology and Counselling, Institute of Health and Biomedical Innovation (IHBI), Queensland University of Technology, GPO Box 2434, Brisbane, Queensland, 4001, Australia

**Keywords:** Online health information, Child health, Child health information seeking, Theory of planned behaviour, Risk taking, Group norm, Parental decision making, Internet use

## Abstract

**Background:**

The quantum increases in home Internet access and available online health information with limited control over information quality highlight the necessity of exploring decision making processes in accessing and using online information, specifically in relation to children who do not make their health decisions. The aim of this study was to understand the processes explaining parents’ decisions to use online health information for child health care.

**Methods:**

Parents (N = 391) completed an initial questionnaire assessing the theory of planned behaviour constructs of attitude, subjective norm, and perceived behavioural control, as well as perceived risk, group norm, and additional demographic factors. Two months later, 187 parents completed a follow-up questionnaire assessing their decisions to use online information for their child’s health care, specifically to 1) diagnose and/or treat their child’s suspected medical condition/illness and 2) increase understanding about a diagnosis or treatment recommended by a health professional.

**Results:**

Hierarchical multiple regression showed that, for both behaviours, attitude, subjective norm, perceived behavioural control, (less) perceived risk, group norm, and (non) medical background were the significant predictors of intention. For parents’ use of online child health information, for both behaviours, intention was the sole significant predictor of behaviour. The findings explain 77% of the variance in parents’ intention to treat/diagnose a child health problem and 74% of the variance in their intentions to increase their understanding about child health concerns.

**Conclusions:**

Understanding parents’ socio-cognitive processes that guide their use of online information for child health care is important given the increase in Internet usage and the sometimes-questionable quality of health information provided online. Findings highlight parents’ thirst for information; there is an urgent need for health professionals to provide parents with evidence-based child health websites in addition to general population education on how to evaluate the quality of online health information.

## Background

In determining how to best care for their child’s health, parents use a range of information sources including general practitioners, books/magazines, family and friends, nurses, pharmacists, alternative practitioners, and increasingly the Internet [1,2]. Khoo et al. [1] found that 43% of Australian parents had sought child health information online, with higher rates reported in other developed countries [3]. The increase in Australian household Internet access from 16% in 1998 to 73% with broadband access in 2011 may be associated with the seemingly parallel increase in parents using online child health information [4,5].

The rapid increase in, and use of, online health information has no corresponding increase in the quality of available material which can be biased and there is little control over the timeliness of updates [6-8]. For example, a systematic review of websites offering advice on acute otitis media treatments identified 41% of sites still recommending antibiotics while only 31% recommended the updated guideline of ‘watch and wait’ [9]. Receiving conflicting information makes it difficult for parents to know how to care for their child, creating confusion, uncertainty, and anxiety about best practice [2]. Considering the dubious quality of some available online information, parent-reported actions following accessing online health information are potentially concerning. Parents diagnose (43%) and treat (33%) child health care using online information, with 18% of Australian parents altering their child’s health management to align with online information [10]. Younger persons, aged between 20 and 35 years, are more likely to report these behaviours, with more women than men likely to engage in seeking health information online [11]. A more recent Australian study found the Internet to be the least trusted child health information source, reported by 9% of parents [1]. The incongruence in these studies requires further exploration.

People use online health information for a range of reasons. These include feeling rushed and receiving limited general lifestyle guidance when seeking advice from doctors; finding information that is more up-to-date, readily accessible, finding alternative treatment options, and to extend their understanding of a health issue before or after a medical consultation [1,12,13]. In particular, the empirical literature provides some evidence describing parents’ reasons for searching and seeking online health information. These include worrying about their child’s health [14], seeking specific information about their child’s health issues (e.g., [15-17] and for self-diagnosis for themselves and their children [18]. However, rather than the previous focus on identifying differences between users and non-users, sites explored, and types of information sought, there is a need to examine systematically the processes underlying parents’ decisions to use online health information for their child’s health care. This study addresses this gap in the literature by using the Theory of Planned Behaviour (TPB) [19] to understand the determinants of parents’ decisions to use online health information to diagnose and/or treat their child’s health issues and to increase understanding about their child’s diagnosis or treatment.

### The theory of planned behaviour

The TPB is a well-validated model [20] that articulates the cognitive determinants of people’s decision making. The TPB specifies intentions as the most proximal determinant of behaviour. Intentions are influenced by attitude (positive or negative evaluation of behaviour), subjective norm (perceived social pressure/acceptance for behavioural performance), and perceived behavioural control (PBC) (perceived ease or difficulty of performing behaviour; also thought to directly predict behaviour). Attitude, subjective norm, and PBC are informed by underlying behavioural, normative, and control beliefs, respectively [19]. Several studies have used the TPB to understand parents’ child health care decisions (e.g., [21,22] but none were identified which use the TPB to explain parents’ decisions to use online information for their child’s health care.

### Perceived risk

Despite strong support for the TPB, a large proportion of variance remains unexplained leading researchers to propose the addition of other theoretically relevant variables to help explain people’s decision-making. Given the potential for unreliable or biased information to be presented online [7,8], errors in parents’ judgement may harm their child’s health [23]. Accordingly, there may be risks associated with using online information, especially if using the information to diagnose and/or treat child health care issues. Similarly, although it may be considered a less risky behaviour because a diagnosis or treatment has been provided by a health professional, using online health information to increase understanding about a child’s diagnosis or treatment still involves the risk that the information found is not reputable, out-of-date, or inaccurate. Given the added value of risk perceptions to the TPB, e.g., [24], and that using online information for child health care may be considered risky behaviour, we included risk perceptions as an additional construct to investigate in this context.

### Group norm

The normative influence of relevant social groups is another important influence on parenting behaviour [25]. In contrast to subjective norm in the TPB, which focuses on perceived social pressure from a range of important referents to perform the behaviour [19], group norm derived from a social identity/self-categorization perspective [26,27] refers to the explicit or implicit prescriptions regarding one’s appropriate attitudes and behaviours as a member of a specific reference group in a specific context [24,28]. Thus, group norm infers that a person’s perceptions about whether other group members perform the behaviour themselves and think it is a good thing to do will influence his or her intentions [28,29]. The normative influences of important others (e.g., friends, other mothers) have been identified by several studies as a key determinant of parenting health practices (e.g., [21,30]. Internet sites (e.g., parenting forums) may provide an avenue for connecting with an online community where a common interest draws parents together, providing information, support, and advice [31,32]. Madge and O’Connor [14] suggest that using the Internet for health information seeking is positively viewed within parenting social groups and important in forming connections with other parents. Thus, group norms may influence parents’ online information seeking behaviour and, as such, the influence of the perceived actions and attitudes of an important referent group (i.e., most mothers I know) was examined specifically in this study.

### The present study

This study was part of a larger project investigating factors that influence parents’ online information seeking behaviours. The TPB, with perceived risk and group norm, as well as socio-demographic factors were used to determine predictors of parents’ intentions and behaviours related to using online health information for their child’s health care. The target behaviours of using child health information from the Internet to 1) diagnose and/or treat their child’s health issues and 2) increase understanding about their child’s diagnosis/treatment from a health care provider were chosen based on previous research outlining these as the online health seeking behaviours of parents [16-18] and piloting work prior to this study (see Methods).

### Hypotheses

From a TPB perspective, it was hypothesised that attitude, subjective norm, and PBC would predict parents’ intention to perform each target behaviour (Hypothesis 1), and intention and PBC would predict actual performance of each target behaviour (Hypothesis 2). For the additional constructs, it was expected that perceived risk and group norm would predict parents’ intentions to use online health information for their child’s health care in relation to each target behaviour (Hypothesis 3). Furthermore, in an exploratory manner, socoiodemographic factors of age, number of children, employment status, education level, medical background, and approximate number of hours spent using the Internet per week were examined to determine if they contributed to the prediction of intentions and actual behaviour for each target behaviour (Hypothesis 4).

## Methods

### Design

An online crossectional, longitudinal study was conducted with two waves of data collection.

### Setting

During March to September, 2010, Australian parents who were current Internet users and had at least one child aged 6 months to 10 years were invited via online parenting and health newsletters and forums, university email groups, and snowballing to complete an online survey (response rate unable to be calculated). An upper age limit of 10 years was used because children aged 10 years and older may search online for health information themselves [33].

### Ethical approval

Ethical approval was obtained from the Queensland University of Technology Human Research Ethics Committee. Participants were parents of young children; however, there was no contact with either the parent or children during recruitment or phase one of the study. Those interested in participating in phase two were asked to indicate their preferred method for follow-up data collection and supply contact details for either email of telephone follow-up. This information, entered into a separate area of the questionnaire, was kept separate throughout the research process.

### Participants

A total of 391 Australian parents (372 mothers, 19 fathers) ranging in age from 22 to 67 years (*M* = 34.96 years; *SD* = 5.73) completed the first survey. Two months later, 187 parents (182 mothers, 5 fathers) ranging in age from 23 to 48 years (*M* = 35.30 years; *SD* = 5.21) completed a follow-up survey to assess their use of online information to diagnose/treat and understand their child’s health concern/s in the previous two months (48% response rate). Table 1 presents sociodemographic characteristics for participants at each time point.

**Table 1:** Sociodemographic characteristics of participants at time 1 and follow up

		**Time 1**	**Follow-up**
		**(*N *= 391) %**	**(*N *= 187) %**
Parent sex (T1 *n* = 391; T2 *n* = 187)	Male	4.9	2.7
	Female	95.1	97.3
Age of parent (T1 *n* = 389; T2 *n* = 187)	18-25	3.8	3.7
	26-35	51.9	47.6
	36-45	40.3	46.0
	46-55	3.8	2.7
	Over 55	0.2	0.0
Number of children^‡^ (T1 *n* = 390; T2 *n* = 187)	1 child	33.1	34.2
	2 children	46.2	44.4
	3 children	14.9	13.9
	4 or more children	5.8	7.5
Age of child 1^±^ (T1 *n* = 389; T2 *n* = 186)	Age 1	0.0	0.0
	Age 2	3.1	0.0
	Age 3	12.3	15.6
	Age 4	11.0	11.3
	Age 5	10.7	12.4
	Age 6	8.4	8.6
	Age 7	10.7	9.1
	Age 8	8.4	7.5
	Age 9	7.7	5.9
	Age 10	4.6	4.8
Age of child 2^±^ (T1 *n* = 257; T2 *n* = 118)	Age 1	0.0	0.0
	Age 2	9.3	11.0
	Age 3	12.8	12.7
	Age 4	13.6	12.7
	Age 5	12.1	11.9
	Age 6	10.9	6.8
	Age 7	8.9	10.2
	Age 8	8.6	6.8
	Age 9	6.2	7.6
	Age 10	5.8	5.9
zAge of child 3^±^ (T1 *n* = 79; T2 *n* = 39)	Age 1	0.0	0.0
	Age 2	13.9	15.4
	Age 3	11.4	10.3
	Age 4	19.0	15.4
	Age 5	13.9	17.9
	Age 6	12.7	10.3
	Age 7	5.1	2.6
	Age 8	5.1	7.7
	Age 9	3.8	5.1
	Age 10	2.5	2.6
Age of child 4 or more^±^ (T1 *n* = 30; T2 *n* = 14)	Age 1	3.0	7.1
	Age 2	10.0	7.1
	Age 3	13.3	7.1
	Age 4	20.0	28.6
	Age 5	6.6	14.3
	Age 6	13.3	0.0
	Age 7	3.0	7.1
	Age 8	10.0	14.3
	Age 9	0.0	0.0
	Age 10	6.6	0.0
Relationship status (T1 *n* = 390; T2 *n* = 186)	Single	3.1	3.2
	In a relationship	1.8	2.1
	Married	75.8	73.7
	Defacto	14.4	15.1
	Divorced/separated	4.4	5.4
	Widowed	0.5	0.5
Location (T1 *n* = 342; T2 *n* = 187)	Northern Territory	0.3	0.5
	Australian Capital Territory	1.5	2.1
	New South Wales	9.3	8.6
	Victoria	4.4	3.7
	Queensland	79.2	77.0
	South Australia	3.5	4.8
	Western Australia	0.6	2.2
	Tasmania	1.2	1.1
Employment status^†^ (T1 *n* = 388)	Full-time	23.7	-
	Not full-time	76.3	-
Education status^†^ (T1 *n* = 387)	University educated	56.1	-
	Not university educated	43.9	-
Medical background^†§^ (T1 *n* = 387)	Yes	19.9	-
	No	80.1	-
Hours Internet use per week (T1 *n* = 385; T2 *n* = 184)	Mean	15.74	26.32
	SD	12.71	32.11
	Median	12.00	15.00
	Mode	10.00	10.00
	Range	1-100	1-200

^†^ Measured at Time 1 only.

^‡^ Children were aged between 6 months and 10 years. An upper age limit of 10 years was used because children aged 10 years and older may search online for health information themselves (Greenfield and Yan, 2006).

^±^ Percentages do not add up to 100% due to parents indicting that they also had other children older than 10 years of age.

^§^ The 77 Participants with a yes response for medical background were those who self-identified their occupation as a nurse (63.6%), midwife (6.5%), medic (soldier) (1.3%), pharmacist (2.6%), dietician (3.9%), nutritionist (2.6%), optometrist (1.3%), physiotherapist (3.9%), occupational therapist (2.6%), radiographer (1.3%), medical scientist (2.6%), or other health professional (7.8%).

T1 = Time 1; T2 = Time 2 (follow up).

Pearson chi-square tests of independence revealed no significant differences between those who responded at Time 1 only and those who responded at both time points on the sociodemographic variables of gender, number of children, relationship status, medical background, education, employment status, and location. T-tests for independent means revealed no significant difference on age between Time 1 responders and responders at both time points. There was a statistically significant difference in the average number of hours spent using the Internet between groups, with Time 1 responders spending less time using the Internet on average (*M* = 15.74, *SD* = 12.71) than responders at both time points (*M* = 26.32, *SD* = 32.11), *t* (567) = −5.61, *p* < .001, η^2^ = .01. A multivariate analysis of variance revealed no significant differences on any of the main study constructs from the Time 1 survey between those who did and did not complete both surveys, *F* (12, 341) = 1.29, *p* = .220.

### Instruments

The Time 1 survey assessed the TPB measures (i.e., attitude, subjective norm, PBC, intention), perceived risk, group norm, and sociodemographics. The follow-up survey administered two months later assessed sociodemographic variables and the decisions parents made in the previous two months to 1) diagnose and/or treat their child’s suspected medical condition/illness (referred to as *diagnose/treat*) and 2) increase understanding about a diagnosis or treatment recommended by a health professional (referred to as *increase understanding*). These two target behaviours were derived from the elicitation study, an earlier part of this overall study with 23 Australian parents (2 fathers, 21 mothers; *M*_*age*_ = 35.35 years, *SD* = 4.31, Range = 29–45 years) who were current Internet users and had at least one child aged between 6 months and 6 years. Upon completion of the interview parents were invited to pilot the developed instrument for face and content validity and readability ease. Seven mothers (*M*_*age*_ = 38.14 years, *SD* = 4.53, Range = 30–42 years) reviewed the instrument with positive comments re readability and importance of the content.

A definition for child health information was included in the surveys for each target behaviour. For *diagnosis/treat*, the following definition was provided: “Any information that you may find online that helps you to make a decision about identifying (e.g., symptoms) and/or treating an illness or medical condition that you believe your child may have (e.g., fever, rash, runny nose)”. For *increase understanding*, the following definition was given: “Any information that you may find online that helps you to understand or find extra information about a diagnosed medical condition/illness (e.g., how long a disease takes to progress) and/or a treatment recommended by a health professional (such as a doctor) (e.g., side effects of medication, alternative treatment options)”.

#### Time 1 survey measures

Measures of the TPB variables [19], perceived risk [34,35], and group norm [36] for *each* of the target behaviours of ‘diagnose/treat’ and ‘increase understanding’ were assessed at Time 1. Measures of self-reported behaviour for each of the target behaviours were obtained at follow up. All items for each of the target behaviours were measured on seven-point response scales, scored 1 (*strongly disagree*) to 7 (*strongly agree*) and coded so that higher values reflected higher levels on the variable under examination, unless otherwise specified.

Intention*.* Three items assessed intention for each behaviour (e.g. “I intend to [target behaviour]”). Items were averaged to create reliable scales (diagnose/treat α = .91; increase understanding α = .89).

Attitude*.* Three semantic differential items assessed attitude for each behaviour (e.g. “[target behaviour] would be 1 *good* to 7 *bad*”, subsequently reverse scored for analyses). Items were averaged to form reliable scales (diagnose/treat α = .97; increase understanding α = .89).

Subjective norm*.* Two items comprised the subjective norm measure for each behaviour (e.g. “Most people who are important to me would support/approve of me [target behaviour]”). Subjective norm items were correlated at *r* (360) = .77, *p* < .001 for diagnose/treat, and *r* (358) = .66, *p* <.001 for increase understanding.

Perceived behavioural control*.* Two items for each behaviour measured PBC (e.g. “It is mostly up to me whether I [target behaviour]”). The PBC items were correlated at *r* (357) = .32, *p* < .001 for diagnose/treat, and *r* (357) = .52, *p* <.001 for increase understanding.

Perceived risk. Two items measured perceived risk for each behaviour (e.g. “What do you think the chances are of you harming your child if you [target behaviour]”, scored 1 *chance is extremely low* to 7 *chance is extremely high*). Risk items were correlated at *r* (359) = .60, *p* < .001 for diagnose/treat, and *r* (359) = .51, *p* <.001 for increase understanding.

Group norm. A pilot study of 23 Australian parents revealed that ‘other mothers’ were an appropriate reference group for the target behaviours. Two items for each behaviour measured group norm (e.g. “Most other mothers that I know [target behaviour]”). Group norm items were correlated at *r* (356) = .87, *p* < .001 for diagnose/treat, and *r* (341) = .81, *p* <.001 for increase understanding.

#### Time 2 measures

Behaviour*.* Parents’ behaviour was measured with two items for each behaviour, “In the past 2 months I have [target behaviour]”, scored 1 *strongly disagree* to 7 *strongly agree*; “In the past 2 months how often did you [target behaviour]?”, scored 1 *never* to 7 *always*. The two items for each behaviour were averaged. Items were significantly correlated at *r*(181) = .75, *p* < .001 for diagnose/treat, and *r* (181) = .78, *p* < .001 for increase understanding.

### Statistical analyses

Means, standard deviations, and bivariate correlations were examined to determine the interrelationships between the TPB variables, perceived risk, and group norm. Bivariate correlations between intentions to perform each target behaviour, and bivariate correlations between actual performance of each behaviour were examined also. A series of hierarchical multiple regression analyses were conducted to predict 1) parent intentions to perform each target behaviour of using child health information from the Internet to ‘diagnose/treat’ and ‘increase understanding’, and 2) actual performance of each target behaviour in a two-month period. For each HMR predicting intentions, the TPB variables were entered at Step 1; group norm and perceived risk at Step 2; and sociodemographic variables of age, number of children, medical background (yes/no), employment status (full time/not full time), education (university educated/not university educated), and approximate hours spent using the Internet per week at Step 3. For each hierarchical multiple regression predicting behaviour, intention and PBC were entered at Step 1; attitude, subjective norm, perceived risk, and group norm at Step 2; and sociodemographic variables at Step 3.

## Results

### Descriptive statistics and correlation matrix

Means, standard deviations, and correlations are reported in Table 2. The bivariate correlation between intentions to perform each target behaviour (*r* (361) = .36, *p* < .001) as well as the bivariate correlation between actual performance of each behaviour (*r* (181) = .59, *p* < .001) showed that, although the target behaviours were related, they were not identical.

**Table 2:** Means, standard deviations, and bivariate correlations for the diagnose/treat and increase understanding TPB variables (attitude, subjective norm, PBC), perceived risk, group norm, intention and behaviour

**Variable**	**1.**	**2.**	**3.**	**4.**	**5.**	**6.**	**7.**	**8.**	**9.**	**10.**	**11.**	**12.**	**13.**	**Mean**	**SD**
1. Age	-	.14**	.05	.15**	.17**	.04	-.06	.17**	-.13*	.09	-.13*	-.13*	-.15*	34.96	5.73
2. Number of children	.14**	-	.09	-.08	-.13*	.14**	.08	.10	.02	-.08	.05	.04	-.04	-	-
3. Medical background	.05	.09	-	.03	.18***	-.11*	-.08	-.08	-.01	-.03	-.02	-.13*	-.10	-	-
4. Employment	.15**	-.08	.03	-	.16**	-.01	-.08	-.06	-.13*	.13*	-.00	-.10	-.09	-	-
5. Education	.17**	-.13*	.18***	.16**	-	-.21***	-.02	-.00	-.03	-.02	.08	.02	.02	-	-
6. Hours/wk Internet	.04	.14**	-.11*	-.01	-.21***	-	.07	.07	.15**	-.09	.08	.13*	.01	15.74	12.71
7. Attitude	-.06	.14**	-.08	-.04	.00	.19***	-	.55***	.35***	-.55***	.25***	.64***	.13	6.47	0.80
8. Subjective norm	.03	.14**	-.05	-.05	.00	.16**	.71***	-	.52***	-.48***	.42***	.77***	.28***	5.56	1.10
9. PBC	-.03	.10	.02	.02	-.01	.11*	.42***	.45***	-	-.41***	.26***	.63***	.15*	6.11	0.85
10. Risk	-.06	-.14**	-.02	.08	-.02	-.16**	-.72***	-.69***	-.41***	-	-.17**	-.57***	-.10	2.18	1.16
11. Group norm	.04	.10	.05	.04	.02	.11*	.58***	.70***	.36***	-.53***	-	.41***	.15*	5.53	1.26
12. Intention	.02	.09	-.13*	-.04	-.04	.21***	.78***	.82***	.46***	-.71***	.64***	-	.36***	5.94	1.04
13. Behaviour	-.08	.02	-.13	-.05	.01	.04	.31***	.37***	.16**	-.26***	.26***	.34***	-	3.72	2.14
Mean	34.96	-	-	-	-	15.74	4.34	3.98	5.17	3.70	4.40	4.12	2.48		
SD	5.73	-	-	-	-	12.71	1.65	1.42	1.18	1.51	1.53	1.55	1.60		

*Note.* Correlations below the diagonal relate to diagnose/treat. Correlations above the diagonal relate to increase understanding.

*Note.* Mean scores on 7-point scales (1–7; higher scores stronger agreement, more important). *Note.* PBC = perceived behavioural control.

*p < .05. **p < .01. ***p < .001.

### Regression analyses predicting intention

A hierarchical multiple regression analysis predicting parents’ intentions to use child health information from the Internet to *diagnose/treat* their child’s suspected medical condition/illness showed that the Step 1 TPB variables accounted for 76% of the variance in intentions, *F*(3, 352) = 365.71, *p* <.001. Perceived risk and group norm at Step 2 explained an additional 1% of the variance, *Fchange*(2, 350) = 6.31, *p* = .002. Sociodemographic variables in Step 3 explained a further 1% of the variance, *Fchange*(6, 344) = 3.65, *p* = .002. At the final step, significant predictors of intentions were attitude, subjective norm, PBC, (less) perceived risk, group norm, (non) medical background, and hours spent per week using the Internet (Table 3).

**Table 3:** Hierarchical regression analyses testing the predictors of parents’ intention and behaviour to use child health information from the Internet to diagnose/treat and increase understanding

		**Diagnose/Treat**					**Increase Understanding**				
	**Variable**	**Step 1β**	**Step 2 β**	**Step 3 β**	**R**^**2**^	**R**^**2**^**Δ**	**Step 1 β**	**Step 2 β**	**Step 3 β**	**R**^**2**^	**R**^**2**^**Δ**
*Intention*											
1	Attitude	.38***	.31***	.28***	.76	.76***	.28***	.23***	.22***	.73	.73***
	Subjective norm	.52***	.45***	.43***			.46***	.41***	.41***		
	PBC	.06*	.05	.06*			.31***	.28***	.27***		
2	Risk		-.12**	-.13**	.77	.01**		-.12**	-.13***	.74	.01**
	Group norm		.07*	.09*				.08**	.08**		
3	Age			-.01	.78	.01**			.02	.75	.01**
	Number of children			-.04					-.04		
	Medical background			-.09**					-.09**		
	Employment			.01					-.02		
	Education			-.02					.04		
	Hours/wk Internet			.05*					.03		
*Behaviour*											
1	Intention	.31***	.30*	.29*	.15	.15***	.41***	.37**	.36**	.12	.12***
	PBC	.02	.02	.01			-.11	-.11	-.10		
2	Attitude		-.06	-.05	.16	.01		-.07	-.08	.12	.01
	Subjective norm		.18	.17				.11	.11		
	Risk		.05	.02				.01	-.01		
	Group norm		.00	.01				.00	-.00		
	Age			-.07	.18	.03			-.12	.16	.03
	Number of children			.00					-.02		
	Medical background			-.13					-.07		
	Employment			-.01					-.03		
	Education			.08					.05		
	Hours/wk Internet			-.02					-.02		

*p < .05. **p < .01. ***p < .001.

For parents’ intentions to use child health information from the Internet to *increase understanding* about a diagnosis or treatment recommended by a health professional, the TPB predictors at Step 1 explained 73% of the variance, *F*(3, 343) = 302.88, *p* <.001. At Step 2, perceived risk and group norm increased the explained variance by 1%, *Fchange*(2, 341) = 8.92, *p* < .001. Sociodemographic variables in Step 3 explained a further 1% of the variance in intentions, *Fchange*(6, 335) = 2.73, *p* = .013. At the final step, attitude, subjective norm, PBC, (less) perceived risk, group norm, and (non) medical background were the significant predictors of intention.

### Regression analyses predicting behaviour

A regression analysis predicting parents’ use of child health information on the Internet to *diagnose/treat* their child’s suspected medical condition/illness showed that intention and PBC at Step 1 explained 15% of the variance in behaviour, *F*(2, 172) = 14.99, *p* <.001. The addition of the Step 2 (*Fchange*(4, 168) = 0.48, *p* = .750) and Step 3 (*Fchange*(6, 162) = 0.85, *p* = .533) variables did not significantly increase the explained variance. At the final step, intention was the only significant predictor of behaviour (Table 3).

For parents’ use of child health information on the Internet to *increase understanding* of a diagnosis/treatment for their child from a health professional, the Step 1 variables explained 12% of the variance in behaviour *F*(2, 173) = 12.27, *p* <.001. The addition of the Step 2 (*Fchange*(4, 169) = 0.41, *p* = .804) and Step 3 (*Fchange*(6, 163) = 0.80, *p* = .573) variables did not significantly increase the explained variance. At the final step, intention was the sole significant predictor of behaviour.

## Discussion

This is one of the first studies to use an established theoretical framework, the TPB, to investigate the psychosocial and demographic factors associated with parents’ use of online information to either 1) diagnose and/or treat their child’s suspected medical condition/illness or 2) increase understanding about a diagnosis or treatment recommended by a health professional. The findings highlight the necessity for health professionals to direct parents to appropriate evidence-based websites; parents’ seek online information, health professionals need to prevent unnecessary harm.

More specifically, the study has revealed that parents were more likely to use online information to increase their understanding about a diagnosis or treatment (than to diagnose and/or treat their child’s health issues. Interestingly, the same pattern of results was revealed for both target behaviours with the TPB variables of attitude, subjective norm, and PBC predicting intention (supporting H1); and intention, but not PBC, predicting behaviour (partially supporting H2). In general, the findings are consistent with other TPB-based studies examining parent–child health care behaviours [21,30]. The finding that PBC was not a significant predictor of parents’ behaviour is consistent with Ajzen’s [19] proposal that PBC becomes less useful in predicting behaviour as volitional control over behaviour increases. It is possible that parents are not accurate in judging how much control they actually have over using online information for their child’s health issues due to factors outside of the parents’ control, such as the information requested not being available or easy to comprehend.

The additional variables of perceived risk and group norm predicted intentions (supporting H3). In support of these findings, despite the dearth of literature exploring factors predicting parents’ internet use, some studies have found worry to be a predictor of internet use in parents of a child with encopresis [37]. Further, parents of a child who had suicided who were internet users or experiencing greater depression who experienced greater stigmatisation from their families, obtained valuable support from online groups [38]. The current study revealed also that parents who did not have a medical background were more likely to intend to engage in these behaviours (partially supporting H 4). For intention to diagnose/treat, those parents reporting using the Internet more were also more likely to intend to use child health information. Overall, the psychosocial determinants identified in this study help to understand parents’ decisions to use online information for their child’s health care. Parents with a more positive attitude toward using online information, who perceive greater social pressure/support to use this information, believe they have greater control and that there are lower risks associated with the behaviours, perceive other mothers have similar attitudes and behaviours and have limited medical experience will have stronger intentions to use online information for child health care. Furthermore, parents with stronger intentions to use online information to diagnose/treat their child’s health issues and to increase understanding about their child’s diagnosis/treatment are more likely to actually do so.

These findings can direct the development of methods for informing parents about appropriate websites and developing educational resources to guide appropriate online help-seeking actions among parents. Initially, there is a need to challenge parents’ attitudes toward accessing online child health information, alert them to the need for caution before acting on online information. There are developed methods for evaluating online health information. For example, Golterman and Banasiak [39] report a framework for evaluating the quality of online health information with strategies for accessing reliable child health sources. They report the importance of alerting Internet users, parents in this instance, to look for the HONCode on child health sites, a Code of Conduct for Medical and Health Web Sites developed by the Health On the Net Foundation [40]. Or search through the toolbar on their website http://www.hon.ch/ to find credible, reliable child health information. Alerting parents and colleagues to the existence of this code and to recommend parents to check for it and/or search for child health information from government, hospital and educational institutions is an important aspect of care in the 21^st^ Century where health consumers can be more informed that health care providers. Additionally, it is important to establish the E-Health literacy of parents prior to referring them to a website; the internet is not an appropriate health information resource for all [13].

Additionally, normative factors were important in informing intentions. Thus, challenging normative beliefs about the perceptions of the approval of important others (such as partners, doctors) and the support from other mothers (mother’s group) for engaging in these target behaviors may also be useful. Parents reported approval toward their intentions to search online; however, normative beliefs were not an independent predictor of behaviour. Mass media campaigns have successfully challenged and changed Australian parents’ sun protective [41] and smoking behaviours [42]. There is a need for a similar approach to child health information that could challenge normative influences toward online child health information. Producers and managers of credible, reliable online child health information, such as hospital and government sites, must be challenged to ensure their sites are the first to appear through Google and/or similar search engines.

Despite parents’ perception of control over using online child health information it had little impact on the variance of their intentions or behaviours. Poor website design or content and the number of advertisements and/or distractors were identified as barriers to using these sites. Online information can be frightening, as described by parents of children with cancer who were afraid of what they may find out [43]. Interestingly, these parents sought online social support rather than information. Health professionals, highlighting the questionable quality of information from sites such as these, can use these factors as a tool when guiding parents toward reputable and appropriate child health websites.

Finally, health professional and media focus on evaluations of the risk involved may help combat any undesired consequences on a child’s health as a result of using online information for child health care. For example, media reports of meningococcal disease send parents to seek online and medical advice for childhood rashes. Medical reports and testimonials from parents, and evidence from the empirical literature, of the consequences of a misdiagnosis based on online information on health outcomes may prompt parents to question their use of Internet-based information to self-diagnose their child’s health issues.

The research has a number of strengths including the use of a well-validated theoretical framework to prospectively examine an important and topical parental behaviour, a consideration of the impact of a range of covariates, and a reasonable sample size. The current study has a number of limitations. The use of self-report data may facilitate socially desirable responses. Further, given that almost one fifth of the sample reported having a medical background, self-selection basis may be an issue to consider as these participants are perhaps more likely to be aware of the limitations of online health information and have a greater ability to find high quality information thus negating some of the risks outlined as a result of the study’s findings. In addition, as no response rate was able to be obtained, it could be presumed that the study recruited a biased sample of respondents; thus, caution should be undertaken in interpreting the generalizability of the findings of the study. Another potential limitation is the use of the self-report behaviour items which may not be the most appropriate measure to determine how often the internet was used to look for information to manage a child’s health care or diagnose/treat. As such, a more objective way to measure internet usage in this context may be a consideration for future investigations. The sample was predominately female and, hence, the relevance of the findings to fathers and extended family and carers is uncertain, although women rather than men are more likely to engage in seeking health information online [11] and are usually the primary caregivers for their child’s health. Finally, although the models in the current study explained a substantial amount of variance in parents’ intentions, a large proportion of variance in the target behaviours remains unexplained.

Future research, then, should consider other variables which might predict parental use of online information seeking behaviour for child health care, such as a consideration of different aspects of risk (e.g., perceived vs. objective risk) in the decision-making process (see [34]). It may be useful for future research, in addition to investigating other variables of interest in this context, to investigate also how information seeking behaviours and use patterns are determined based on condition. For example, the information sought for cancer might be different than asthma, thus highlighting the need for a more targeted approach based on the information being sort for a particular medical condition.

Parents reported greater control over seeking online child health information to increase their understanding than in using it to diagnose/treat their child. This is an area where health professionals are found lacking. Today, they also have an important role in not only providing parents with accurate information and ensuring their understanding of this information. They are also charged with the need to assist parents in their online searching by providing them with web addresses of sites reporting evidence-based child health information, and/or specific websites to address their own child’s health needs. From a health professional perspective it is reassuring that parents had less control, though some did still report control, over diagnosing/treating their child with web-based information.

## Conclusion

Overall, we found support for the efficacy of the TPB constructs, attitude, subjective norm and perceived behavioural control; and the role of perceived risk and group norm in understanding parents’ decisions to use online information to increase understanding for a child’s diagnosis and treatment and to diagnose and/or treat a child’s health care issues. Understanding parents’ attitudes and beliefs about these online child health care behaviours is important given the increase in Internet usage and the sometimes-questionable quality of health information provided online which may potentially lead to grave health consequences for children through parental misdiagnosis. Further research is needed to identify specific information needed by parents to understand their child’s illness/developmental concerns. However, in the interim health practitioners are obliged to not only provide parents with appropriate verbal education about their child’s health but also to provide them with written information and appropriate websites to extend this information if they so desire. Initially health practitioners could follow the approach used by pharmacists in Australia; giving clients a printed copy of the *drug company*, developed consumer report of drug actions, interactions and side effects as well as directions for use. Parents want more information. We, the health profession, are now charged with not only providing credible, reliable consumer information but also in assessing their E-health literacy and directing parents to additional sources of information and how to access this online.

## Abbreviations

TPB: Theory of Planned Behaviour; PBC: Perceived behavioural control; M: Mean; SD: Standard deviation; HMR: Hierarchical Multiple Regression.

## Competing interests

The authors declare that they have no competing interests.

## Authors’ contribution

AW conceived the study, study design and coordination, article revision. KW study design, article revision. MH data analysis, study coordination, article revision. KH data analysis, article drafting, article revision. All authors read and approved the final manuscript.

## Pre-publication history

The pre-publication history for this paper can be accessed here:

http://www.biomedcentral.com/1472-6947/12/144/prepub

## References

[B1] Khoo K Bolt P Babl FE Jury S Goldman RD Health information seeking by parents in the internet age J Paediatr Child Hlth 2008 44 419 423 10.1111/j.1440-1754.2008.01322.x 18564080

[B2] Walsh A Edwards H Fraser J Influences on parents' fever management: beliefs, experiences and information sources JCN 2007 16 2331 2340 17419783 10.1111/j.1365-2702.2006.01890.x

[B3] Goldman RD Macpherson A Internet health information use and e-mail access by parents attending a paediatric emergency department Emerg Med J 2006 23 345 348 10.1136/emj.2005.026872 16627833 PMC2564079

[B4] Census data http://www.abs.gov.au/websitedbs/D3310114.nsf/home/home.

[B5] Household Use of Information Technology, Australia 2010–11 http://www.abs.gov.au/ausstats/abs@.nsf/mf/8146.0.

[B6] Clauson KA PH Kamel Boulos MN Dzenowagis JH Scope, completeness, and accuracy of drug information in wikipedia Ann Pharmacother 2008 42 1814 1821 10.1345/aph.1L474 19017825

[B7] Scullard P Peacock C Davies P Googling children’s health: reliability of medical advice on the internet. Arch Dis Child 2010 95 580 582 10.1136/adc.2009.168856 20371593

[B8] Zun LS Blume DN Lester J Simpson G Downey L Accuracy of emergency medical information on the web Am J Emerg Med 2004 22 94 97 10.1016/j.ajem.2003.12.009 15011221

[B9] Holland ML Fagnano M Appropriate antibiotic use for acute otitis media: what consumers find using web searches Clinl Pediatr 2008 47 452 456 10.1177/0009922807313274 18310526

[B10] Wainstein B Sterling-Levis K Baker S Taitz J Brydon M Use of the internet by parents of paediatric patients J Paediatr Child Hlth 2006 42 528 532 10.1111/j.1440-1754.2006.00916.x 16925539

[B11] Ybarra M Suman M Reasons, assessments and actions taken: sex and age differences in uses of internet health information Hlth Educ Res 2008 23 512 521 10.1093/her/cyl062 16880222

[B12] Sillence E Briggs P Please advise: using the internet for health and financial advice Comp Human Behr 2007 23 727 748 10.1016/j.chb.2004.11.006

[B13] Knapp C Madden V Wang H Sloyer P Shenkman E Internet use and eHealth literacy of low-income parents whose children have special health care needs J Med Internet Res 2011 13 e75 e75 10.2196/jmir.1697 21960017 PMC3222184

[B14] Magee JC Ritterband LM Thorndike FP Cox DJ Borowitz SM Exploring the relationship between parental worry about their children's health and usage of an internet intervention for pediatric encopresis J Pediatr Psych 2009 34 530 538 10.1093/jpepsy/jsn091 PMC2722136 18772228

[B15] DeLuca J Kearney M Norton S Arnold G Internet use by parents with infants of positive newborn screens J Inherit Metabolic Dis 2012 35 877 8884 10.1007/s10545-011-9449-7 22297410

[B16] Rahi JS Manaras I Barr K Information sources and their use by parents of children with ophthalmic disorders Invest Ophthal & Visual Sci 2003 44 2457 2460 10.1167/iovs.02-1184 12766043

[B17] Semere W Karamanoukian HL Levitt M Edwards T Murero M D'Ancona G Donias HW Glick PL A pediatric surgery study: parent usage of the internet for medical information J Pediatr Surg 2003 38 560 564 10.1053/jpsu.2003.50122 12677566

[B18] Sarkadi A Bremberg S Socially unbiased parenting support on the internet: a cross-sectional study of users of a large swedish parenting website Child: Care, Hlth Develop 2005 31 43 52 10.1111/j.1365-2214.2005.00475.x 15658965

[B19] Ajzen I The theory of planned behavior Orgl Behav Human Decisc Proc 1991 50 179 211 10.1016/0749-5978(91)90020-T

[B20] Armitage CJ Conner M Efficacy of the theory of planned behaviour: a meta-analytic review Br J Soc Psychol 2001 40 471 499 10.1348/014466601164939 11795063

[B21] Walsh A Edwards H Fraser J Attitudes and subjective norms: determinants of parents' intentions to reduce childhood fever with medications Hlth Educ Res 2009 24 531 545 10.1093/her/cyn055 18974070

[B22] Hamilton K Daniels L White K Murray N Walsh A Predicting mothers’ decisions to introduce complementary feeding at 6 months: an investigation using an extended theory of planned behaviour Appetite 2011 56 674 681 10.1016/j.appet.2011.02.002 21316413

[B23] Kiley R Does the internet harm health? some evidence exists that the internet does harm health BMJl 2003 323 238 10.1136/bmj.324.7331.238a PMC1122149 11809659

[B24] White K Hogg M Terry D Improving attitude-behavior correspondence through exposure to normative support from a salient ingroup Basic Appl Soc Psychol 2002 24 91 103

[B25] Abidin RR The determinants of parenting behavior J Clin Child Adolesc Psychol 1992 21 407 412 10.1207/s15374424jccp2104_12

[B26] Hogg M Abrams D Social identification: a social psychology of intergroup relations and group processes 1988 London: Routledge

[B27] Turner J Hogg M Oakes P Rediscovering the social group: a self-categorisation theory 1987 Oxford: Blackwell

[B28] Amiot C Sansfacon S Louis W Yelle M Can intergroup behaviors be emitted out of self-determined reasons? testing the role of group norms and behavioral congruence in the internalisation of discrimination and parity behaviors Personal Soc Psychol Bull 2012 38 63 76 10.1177/0146167211429804 22109251

[B29] Johnston K White K Binge-drinking: a test of the role of group norms in the theory of planned behaviour Psychol Hlth 2003 18 63 77 10.1080/0887044021000037835

[B30] Thomson C White K Hamilton K Investigating mothers’ decisions about their child’s sun-protective behaviour using an extended theory of planned behaviour J Health Psychol 2012 17 1001 1010 10.1177/1359105311433905 22253324

[B31] Baum LS Internet parent support groups for primary caregivers of a child with special health care needs Pediatr Nurs 2004 30 381 15587531

[B32] Burrows R Nettleton S Please N Virtual community care? social policy and the emergence of computer mediated social support Inf Commun Soc 2000 3 1 6 10.1080/136911800359392

[B33] Greenfield P Yan Z Children, adolescents, and the internet: a new field of inquiry in developmental psychology Develop Psychol 2006 42 391 394 10.1037/0012-1649.42.3.391 16756431

[B34] Ranby K Aiken L Gerend M Erchull M Perceived susceptibility measures are not interchangeable: absolute, direct comparative, and indirect comparative risk Hlth Psychol 2010 29 20 28 10.1037/a0016623 20063932

[B35] White K Hyde M Swimming between the flags: a preliminary exploration of the influences on Australians’ intentions to swim between the flags at patrolled beaches Accid Anal Prevent 2010 42 1831 1838 10.1016/j.aap.2010.05.004 20728634

[B36] Terry DJ Hogg MA White KM The theory of planned behaviour: self-identity, social identity and group norms Br J Soc Psychol 1999 38 225 244 10.1348/014466699164149 10520477

[B37] Magee J Ritterband L Thorndike F Cox D Borowitz S Exploring the relationship between parental worry about their children's health and usage of an Internet intervention for pediatric encopresis J Pediatr Psychol 2009 34 530 538 10.1093/jpepsy/jsn091 18772228 PMC2722136

[B38] Feigelman W Gorman B Beal K Jordan J Internet support groups for suicide survivors: a new mode for gaining bereavement assistance Omega 2008 57 217 243 18837172 10.2190/OM.57.3.a

[B39] Golterman L Banasiak NC Evaluating web sites: reliable child health resources for parents Pediatr Nurs 2011 37 81 83 21661608

[B40] HON code of conduct (HONCode) for medical and health websiteshttp://www.hon.ch/.

[B41] Shih ST-F Carter R Sinclair C Mihalopoulos C Vos T Economic evaluation of skin cancer prevention in Australia Prevent Med 2009 49 449 453 10.1016/j.ypmed.2009.09.008 19747936

[B42] Yiow L Australia: campaign gets smoking parents to cut down Tob Control 2005 14 363 363 16319353 PMC1748119

[B43] Gage E Panagakis C The devil you know: parents seeking information online for paediatric cancer Soc Hlth Ill 2012 34 444 458 10.1111/j.1467-9566.2011.01386.x PMC3223539 21854400

